# Predictive markers for anti-inflammatory treatment response in thyroid eye disease

**DOI:** 10.3389/fendo.2023.1292519

**Published:** 2023-12-04

**Authors:** Haiyang Zhang, Jingyuan Fan, Jialu Qu, Qinghe Han, Huifang Zhou, Xuefei Song

**Affiliations:** ^1^ Department of Ophthalmology, Shanghai Ninth People’s Hospital, Shanghai Jiao Tong University School of Medicine, Shanghai, China; ^2^ Shanghai Key Laboratory of Orbital Diseases and Ocular Oncology, Shanghai, China; ^3^ Department of Radiology, The Second Hospital of Jilin University, Changchun, Jilin, China

**Keywords:** thyroid eye disease, anti-inflammatory, intravenous glucocorticoids, treatment response prediction, biomarker

## Abstract

Anti-inflammatory treatment is the primary and vital therapeutic approach for active, moderate-to-severe thyroid eye disease (TED). Accurate pretreatment prediction of treatment response is of paramount importance for the prognosis of patients. However, relying solely on the clinical activity score asa determinant of activity has led to unsatisfactory treatment outcomes. In recent years, significant advancements have been made in identifying predictive markers for anti-inflammatory treatment response in TED, clinical markers, body fluid biomarkers and imaging biomarkers. Several clinical studies have developed prediction models based on these markers. However, there is still a lack of comprehensive elucidation or comparison between the different markers. Therefore, this review aims to provide a detailed analysis of the definition, characteristics, and application of predictive markers for anti-inflammatory treatment response in TED. Through detailed literature search, 26 articles applying anti-inflammatory treatment effect prediction with a total of 1948 TED patients were used for analysis and discussion. By gaining a better understanding of the current research on predictive markers, we can accelerate and guide the exploration of treatment prediction strategies, leading us towards an era of precise therapy for TED.

## 1 Introduction

Thyroid eye disease, also known as Graves’ orbitopathy (GO), is a prevalent inflammatory orbital disease with various accompanying signs, including exophthalmos, lid retractions, periorbital edema, diplopia, and eye movement disorders (1, 2). In more severe cases, exposure keratopathy and dysthyroid optic neuropathy may develop, significantly impacting patients’ visual health and overall quality of life (3). The clinical guidelines established by the European Group on Graves’ Orbitopathy (EUGOGO) provide valuable insights into determining treatment plans based on the assessment of disease activity and severity (1). Evaluation of disease activity by the clinical activity score (CAS) helps categorize TED into an active stage (CAS ≥ 3) or an inactive stage (CAS < 3) and guide therapeutic decision making (4). For patients with active, moderate-to-severe TED, initiating anti-inflammatory treatment serves as the first step in the treatment schedule. These treatments include intravenous glucocorticoid (IVGC), orbital radiation therapy (ORT), periocular triamcinolone acetonide injection, and targeted immunosuppressive agents (i.e., insulin-like growth factor 1 receptor (IGF-1R) monoclonal antibody, interleukin (IL)-6 monoclonal antibody, etc.). All these treatments aim to alleviate inflammation and relieve the disease.

Up till now, CAS remains the predominant method for determining the appropriateness of anti-inflammatory treatment in TED. TED patients with CAS ≥ 3 are defined as active TED patients and are usually prescribed IVGC for disease activity control. ORT and other anti-inflammatory treatment are given as the second line treatment for active patients. However, several clinical studies have revealed that the anti-inflammatory response rate of patients based on pretreatment CAS evaluation is not encouraging. On average, approximately 30% of active, moderate-to-severe patients (CAS ≥ 3) did not benefit from IVGC (5). Similarly, the unresponsive rate for ORT was approximately 15% (6). In a recent study conducted by Jiang et al. (7), it was reported that among patients with CAS ≥ 3, 38% showed no response to IVGC. Conversely, 45% patients with CAS < 3 demonstrated improvement after IVGC. These findings suggest that using CAS for activity assessment and treatment response prediction in TED remains unsatisfactory. Moreover, inappropriate selection of treatment schedules would not only delay the progress of the disease but also give rise to adverse consequences such as hyperglycemia, osteoporosis, and water-sodium retention. Hence, the proper selection of anti-inflammatory therapy and administration protocols for TED treatment holds crucial significance (8).

Building prediction models offers a promising approach in addressing this issue. Prediction models utilize predictive markers derived from clinical-related factors to estimate the probability or likelihood of a specific clinical outcome or event (9). Predictive markers encompass a range of elements that can indicate biological and pathological processes or responses to specific treatments. They have already been widely utilized in the fields of cancer, immune disorders, eye diseases, etc. Related categories include clinical markers, body fluid biomarkers, imaging biomarkers, among others (10). For example, clinical markers (i.e., age, sex, disease history, etc.) are combined for predicting risk of gastric cancer (11); body fluid biomarkers (i.e., anti-DNA titers, complement levels, antiphospholipid antibodies, etc.) are combined for treatment response prediction of lupus nephritis (12); while quantitative analysis of eyes with imaging biomarkers (i.e., infrared imaging, color fundus photography, etc.) are employed for diagnosis and treatment response prediction of macular degeneration (13). In the field of thyroid eye disease, recent years witnessed especial development of predictive markers. Compared with adopting CAS as the only indicator in the past, the combination of clinical and biological markers has contributed markedly to the response prediction ability among different treatment modalities (14). Since each predictive marker owns its inherent advantages and limitations, it is necessary to describe in detail their different functions for predicting anti-inflammatory treatment response in TED.

We conducted an extensive literature review on this subject, with a specific focus on studies that discuss the prediction of responses to anti-inflammatory treatments in patients with TED. Our review identified 26 articles, collectively encompassing 1,948 TED patients, and we compiled the pertinent data into **Supplementary Table 1**. We meticulously summarized the basic information, treatment approaches, evaluation methodologies, and clinical indications from these relevant studies. It is noteworthy that all the studies’ predictive markers can be categorized into three distinct groups: clinical markers (article 1 to 5), body fluid biomarkers (article 6 to 9), and imaging biomarkers (articles 10 to 26). This categorization effectively encapsulates the current landscape of predictive markers for anti-inflammatory treatments in TED. In the subsequent sections, we further elaborate on each of these three categories, delving into the details of clinical markers, body fluid biomarkers, and imaging biomarkers to provide a comprehensive exploration of this intricate issue.

## 2 Clinical markers

Clinical markers refer to readily accessible and observable clinical materials that provide indications of a specific disorder. In the context of TED, clinical markers include demographic information (i.e., age, sex, etc.), clinical signs to determine severity and activity (i.e., NOSPECS, CAS, etc.), environmental factors affecting prognosis of the disease (i.e., smoking status, etc.), and clinical course (i.e., disease or symptom duration, etc.) (15), among others. As a clinical practice-derived scoring system, CAS is widely used in the staging of TED and the selection of treatment methods. Furthermore, various studies demonstrated that smoking status, disease duration and age are independent predictive markers for the response to anti-inflammatory treatment in TED (16–20).

Based on traditional clinical markers mentioned above, researchers have discovered quite a few predictive markers for treatment response to IVGC. By studying the response rate of 90 consecutive patients with moderate-to-severe and active TED, Wang et al. (21) discovered that pretreatment CAS (odds ratio (OR) =1.653, P<0.01) and restoration of euthyroidism (OR=3.282, P=0.039) were positive independent predictors of IVGC treatment response while symptom duration (OR=0.984, P=0.012) was a negative independent predictor. Besides, the combination of them presented a better predictive ability (AUC=0.784). In a study of 159 TED patients, Bartalena et al. (22) found that early CAS (6-week-CAS) can be used to predict the effect of late IVGC (12-week-CAS). The relative risk of no response at week 12 for “unchanged” patients at week 6 was 2.81 for CAS. Ahn et al. (20) discovered that age (OR=0.918, P=0.017) could be an independent predictor for the treatment response to IVGC. Xing et al. (16) found that smoking (OR=12.40, P=0.035) was an independent risk factor for negative treatment response to IVGC, indicating that smokers with TED should be given higher dose of IVGC.

Apart from the most common anti-inflammatory treatment as IVGC, clinical markers can also be applied in ORT response prediction for TED. As early as 1990, Petersen et al. (23) discovered that male gender, advanced age, need for concurrent therapy for hyperthyroidism, and no history of hyperthyroidism were negatively related with ORT response in TED patients. Prabhu et al. (24) later demonstrated that smoking status (OR=3.23) and total symptom score at 4 months (OR=1.59) were independent predictive markers for response to ORT. Similar conclusion was reached by Li et al. (19), proving that smoking status (OR=2.88, P=0.008), together with disease duration (OR=3.33, P=0.017), could be independent predictive markers of ORT response, which was also verified by Oke et al. later (25). However, compared with IVGC, prediction methods for ORT are restricted to independent predictive markers, no effective prediction model has yet been built. Further research is anticipated for developing a response prediction model based on combined markers.

In summary, although there are already many clinical markers for predicting the response to anti-inflammatory treatment in TED, the overall prediction ability of clinical markers is still unsatisfactory due to their inherent limitations. Taking CAS as an example, the limitations include: First, CAS could only assess the visible anterior part of an orbit rather than the deep orbital structure, resulting in an indirect representation of the actual disease status (26). Second, heterogeneity among patients (i.e., races, ages, and skin surface conditions, etc.) were likely to be ignored by the current universal evaluation criteria, which were established by EUGOGO based on Caucasian patients (27). Third, the evaluation outcome of each criterion in CAS is binary, which might not be accurate enough as it reduces precise information. To conclude, though clinical markers demonstrate close relation to disease progress, their individual predictive capability for treatment response is limited. Therefore, future research could focus more on the combined predictability of various clinical markers.

## 3 Body fluid biomarkers

In this section, we explore common body fluid biomarkers associated with TED, mainly found in blood or tear fluid. Regarding blood biomarkers, they play a crucial role in reflecting systemic circulation, including thyroid function and related antibodies, cytokines, immunological indicators, and other metabolites. Tear biomarkers, on the other hand, are indicators of local enrichment, consisting of various cytokines and oxidative stress markers. While urinary biomarkers have been reported in some studies, their clinical application in predicting TED treatment response is limited by challenges in obtaining and measuring 24-hour urine volume accurately. Only a few biomarkers like urinary glycosaminoglycans have been discovered to be useful in detecting inactive stage, with its negative predictive value reaching 96% (28).

### 3.1 Blood biomarkers

Since TED is an autoimmune disease, changes in blood antibodies triggered by the inflammatory lesions lead to a series of changes in blood metabolites. Thyroid function and related antibodies are among the widely detectable and applicable biomarkers. Notable examples include thyrotropin receptor antibody (TRAb), thyroid-stimulating hormone (TSH), thyrotropin-binding inhibitory immunoglobulin (TBII), etc. (29). These markers are correlated with thyroid hormone overproduction and orbital adipose proliferation, thus reflecting the degenerating status of thyroid function during inflammatory progress (1). Besides thyroid function and related antibodies, other reported blood biomarkers in TED comprise a range of cytokines, such as IL-4, IL-6, IL-8, IL-10, IL-16 (30), CXC chemokine ligand (CXCL) (31), specific antibodies (i.e., serum anti-acetaldehyde dehydrogenase 2 antibody (32), etc.), immunological indicators (i.e., active rosette forming cells, total rosette forming cells (33), myeloid dendritic cells (34), etc.). Additionally, various physiological metabolites, including triglyceride (29), serum uric acid (35), low-density lipoprotein cholesterol (LDLc) (36), etc., have also been identified as relevant blood biomarkers. These biomarkers have proven their utility in the diagnosis and activity assessment of TED, while also holding potential predictive ability. Several of the blood biomarkers mentioned above have been proven effective in predicting the response to anti-inflammatory treatment. For instance, Aung et al. (37) found that high levels of free thyroxine (fT4) (OR=1.04, P=0.002) and TRAb (OR=1.03, P=0.004) were two independent predictive clinical markers for ORT response. Ahn et al. (20) found that serum TBII (OR=0.921, P=0.012) could be an independent predictor of IVGC response in TED. Similarly, Hu et al. (29) discovered a negative correlation between pretreatment triglycerides (OR=0.090, P=0.001) and IVGC treatment response. Besides, Mysliwiec et al. (31) revealed that an increased concentration of circulating CXCL9 (p < 0.02) could reflect the activity of orbital inflammation and serve as a predictor for treatment response to IVGC.

In addition to simple adoption of body fluid biomarkers, some researchers have attempted to combine these biomarkers with clinical markers. For instance, Naselli et al. (36) developed a predictive model based on early clinical response (CAS at week 6) (P<0.001) and low-density lipoprotein cholesterol (r=-0.25, P=0.045), which also turned out to be successful (AUC=0.805) in predicting later IVGC response (CAS at week 12). Meanwhile, Hu et al. (29) established a multiple regression model based on pretreatment TRAb level, pretreatment triglyceride level, elevated TSH level, pretreatment CAS, and symptom duration (AUC=0.915). The predictive accuracy of this model was much higher than that of merely using clinical markers. Based on a machine learning model of extreme gradient boosting (XGBoost), Park et al. (38) also constructed a prediction model with both blood biomarkers and clinical markers (AUC=0.861). Among the 17 markers they included, thyroid stimulating immunoglobulin (TSI) (OR=1.005, P=0.038) and the degree of extraocular muscle (EOM) movement limitation (OR=0.463, P=0.014) were the two most significant independent predictors. However, this machine learning model failed to fully explain the changing tendency of TSH level which was contrary to previous studies and deserved further verification.

To conclude, despite the discovery and utilization of many TED-related blood biomarkers, the availability of biomarkers for predicting treatment response remains limited. Some researchers speculate that (39, 40) this phenomenon may be due to several reasons: First, many blood indicators are involved in multiple metabolic processes, which leads to their poor stability and specificity in representing the disease status of a specific structure, like the orbit. Second, indicators well detected at the beginning of the autoimmune process may later be masked by hemodilution or increasing production of other antibodies and cytokines. Third, prominent individual heterogeneity may affect the universality of blood biomarkers. Indeed, though blood biomarkers commonly exhibit a lack of specificity, certain indicators still hold important value and could support prediction models for TED.

### 3.2 Tear biomarkers

Tear fluid, originating from the lacrimal gland and passaging the ocular surface, is a valuable biomarker that reflects both lacrimal gland status and ocular surface-specific alterations. Since TED patients often experience lymphocytic infiltration and fatty hyperplasia in the lacrimal gland tissue, such inflammation can cause changes in tear composition, whose pathological changes would eventually lead to ocular surface damage (41). As reported, 65% to 80% TED patients have experienced symptoms like dry eye (42).

Numerous studies have indicated that tear biomarkers could serve as predictive markers for TED diagnosis. As an autoimmune disease that involves differentiation of helper T lymphocyte-17 and secretion of IL-17, TED triggers a pathogenesis that leads to a notable increase in cytokines such as tumor necrosis factor-α (TNF-α), interleukins (i.e., IL-1β, IL-6, IL-13, IL-17A, IL-18), and regulated upon activation, normal T Cell expressed and presumably secreted (RANTES), etc. (43, 44), the elevation of which eventually contribute to the activity assessment of TED. However, there still lacks evidence supporting the utilization of tear biomarkers in predicting TED treatment response.

Up till now, only hypotheses have been made regarding the potential predictive value of tear biomarkers. Choi et al. (45) found that the increase of oxidative stress markers 8-hydroxydeoxyguanosine and malondialdehyde in tears significantly correlated with CAS changes before and after treatment (r = 0.676, P<0.001; r = 0.506, P=0.002). Xu et al. (46) added that the decrease of IL-6, IL-8 and vascular endothelial growth factor levels were positively correlated with the decrease of CAS at 12 weeks (P=0.015; P=0.018; P=0.049). Moreover, they proposed a new evaluation index based on reduction of ocular surface damage (i.e., dry eye, strabismus, diplopia) for predicting TED treatment response, which offers valuable insights for further studies into tear composition and clinical symptoms.

In summary, current research on tear biomarkers is mainly constrained to stable components such as cytokines and thyroid function related antibodies, while the performance of tear proteins is relatively poor due to its difficulty in extraction and instability during metabolism (47). Nevertheless, the potential significance of tear biomarkers should not be undervalued. As a non-invasive technique, tear sampling endows better advantage in providing longitudinal sampling results, which aligns well with the disease progress (48). Although specific tear biomarkers for treatment response prediction have not yet been discovered, some studies have attempted to compare tear biomarkers with CAS changes before and after treatment, suggesting its promising correlation with orbital inflammation and providing evidence for its potential application in predicting treatment response.

## 4 Imaging biomarkers

In the diagnosis and treatment of TED, orbital imaging enables an intuitive representation of the disease status. Widely applied clinical imaging examinations for TED include magnetic resonance imaging (MRI), nuclide imaging, digital infrared thermal imaging (DITI), computed tomography (CT), ultrasound, etc. These imaging modalities possess diverse features and potential to serve as predictive imaging biomarkers for predicting the response to anti-inflammatory treatment.

### 4.1 MRI

MRI is a powerful imaging technique known for multiparametric images in high spatial resolution, precise differentiation of tissues and fluids, as well as detection of subtle retrobulbar lesions. In terms of objectivity and sensitivity, MRI significantly outperforms CAS in identifying the subtle changes (49). Imaging biomarkers based on MRI have already been widely used in TED for staging, diagnosis, treatment response prediction and efficacy monitoring (39). Commonly utilized MRI sequences in TED include T1-weighted sequences (T1WI), T2-weighted sequences (T2WI), fat-suppressed sequences (FS), iterative secomposition of water and fat with echo asymmetry and least-squares estimation (IDEAL), and diffusion weighted imaging (DWI). Based on these sequences, a series of quantitative imaging biomarkers are utilized to capture specific lesions of TED in EOM, orbital fat, and lacrimal glands (50–54). According to their different derivations, MRI biomarkers could be categorized as morphological and functional imaging biomarkers. Morphological imaging biomarkers refer to the quantitative measurements of specific orbital tissues or sites (i.e., thickness, width, etc.), while functional imaging biomarkers refer to functional measurements of tissue sites (i.e., relaxation time, signal intensity values, signal intensity ratio, etc.).

Regarding morphological imaging biomarkers, Xu et al. (55) successfully predicted the treatment response to IVGC by measuring the thickness of exophthalmos and the ratio of inferior rectus/fat on T1WI (AUC=0.950). Similarly based on T1WI sequences, Hu et al. (52) discovered that the ratio of lacrimal gland herniation to orbital fat thickness could serve as a robust predictive marker (AUC=0.702). In addition to simple measurement of ocular surface features, they later attempted to combine it with functional imaging biomarkers and clinical characteristics. In terms of functional indicators, Utech et al. (56) demonstrated early in 1995 that EOM T2 relaxation time (T2RT) could be a predictive marker for IVGC treatment response (P<0.01), whose conclusion was enhanced by Hu et al. (57) with a similar model (AUC=0.804) based on T2RT_min_ of EOM on T2 mapping (P=0.003). Besides T2RT, signal intensity (SI) of EOM (P=0.008) or orbital connective tissue (P=0.02) on STIR (58), signal intensity ratio (SIR) of EOMs on T2WI (P=0.004) (59), a composite parameter based on orbital fat volumetry and water fraction on T2-FS imaging (right eye, r=0.82; left eye, r=0.79) (60), low SI of EOM on T1 mapping (AUC=0.890) (61), all these imaging biomarkers and the prediction models based on them demonstrated strong predictive value for IVGC treatment response. Furthermore, Lingam et al. (62) demonstrated an association between apparent diffusion coefficient (ADC) of EOMs on DWI and SIR of EOMs on STIR (r = 0.75, P<0.001), providing future potency for incorporating ADC as a predictive marker.

Besides adoption of individual imaging sequence, additional strategies have also been explored to improve the predictive efficacy. Some researchers attempted to combine different MRI sequences or different biomarker types (52, 63), others employed advanced post-processing methods such as radiomics analysis (53, 64, 65). For instance, with combination of different MRI sequences, Zhai et al. (63) constructed a prediction model (AUC=0.844) based on T2RT_mean_ of EOM on T2 mapping and WF_max_ of EOM on T2 IDEAL. By combining imaging biomarkers with clinical markers, Wang et al. (65) constructed a prediction model based on entropy of EOM, uniformity of EOM and disease duration (AUC=0.802). Another worth-noting point in this study is their employment of texture analysis in orbital MRI, which is a prospective and advanced post-processing method.

Among other types of anti-inflammatory treatment, however, MRI biomarker displayed mixed predictive value. In terms of periocular triamcinolone acetonide injection (66), SIR of levator palpebrae superioris on T2WI−FS (P<0.001) turned out to be a positive predictive marker; when it comes to the treatment response prediction of ORT, the performance of MRI biomarkers remains modest. Prummel et al. (67) only observed limited value of the longest T2RT of EOM on T2WI (AUC=0.630) in predicting treatment response to ORT. Similar unsatisfactory result was reached by Ott et al. (68) who discovered unsignificant predictive value of SI of EOM on T1WI (P=0.08). Only one research reached an opposite conclusion, as Ito et al. (69) found that thyroid-stimulating antibody (TSAb) rate (OR=1.010, P<0.001) and standard deviation (SD) of SI of EOM on T2WI (OR=0.974, P<0.001) were independent predictive markers for the 2-year cumulative relapse-free rate after combined IVGC and ORT treatment (AUC=0.910).

In summary, owing to its accurate depiction of subtle retrobulbar lesions, MRI excels at predicting treatment response with better reflection of inflammatory activities. Multiple studies have indicated that combination of quantitative MRI parameters with other clinical predictive markers could enhance the accuracy of the prediction model. However, current application of MRI biomarkers is restricted to predicting treatment response to IVGC, while future research into the prediction of ORT is equally needed.

### 4.2 Nuclide imaging

Nuclide imaging biomarker is based on a technique that tracks inflammatory activity at molecular level. With tracers containing radioactive elements, nuclide imaging provides evidence for diagnosis and treatment of various conditions (i.e., endocrine tumors (70), rheumatoid arthritis (71), etc.). In the field of TED, there has been an expanding utilization of single-photon emission computed tomography (SPECT) and positron emission tomography (PET), with their commonly used tracers including 111Indium-diethylenetriaminepentaaceticacid (DTPA) (72), 99m-Technetium (99mTc)-DTPA (73–75), Gallium-67 citrate (76), Gallium-68-DOTA-NOC (77), etc.

Attempts for predicting response to anti-inflammatory treatment with nuclide biomarkers were made as early as in 1998. Colao et al. (78) first tried to predict treatment response to IVGC with semi-quantitative index of orbital-to-brain ratio obtained by 111Indium-octreotide scintigraphy, but their result failed to reach clinical significance (r = 0.3, P>0.05). It was not until Konuk et al. (76) discovered orbital gallium-67 scintigraphy as a predictive marker (OR=1.74, P<0.001), that subsequent studies developed various independent predictive nuclide biomarkers. These predictive markers include baseline 99mTc-anti-TNF-α scintigraphy uptake region of interest (ROI)-ratios on SPECT/CT (P=0.036) (79), as well as the maximum uptake ratio of four EOMs (U_max_) obtained by 99mTc-DTPA SPECT/CT (OR=2.08, P=0.001) (80), which outperformed CAS among patients with CAS≥3 (AUC=0.850 vs 0.780). For response prediction to ORT, Ujhelyi et al. (81) demonstrated that an elevated orbital DTPA uptake on SPECT (P<0.001) could be an independent predictive marker, whose uptake above 12.28 MBq/cm^3^ could indicate better treatment response with a positive predictive value (PPV) reaching 76%.

In conclusion, while cost and patient accessibility pose current challenges (81), ongoing efforts to enhance SPECT’s availability, coupled with its evolving performance, underscore its potential in predicting responses to anti-inflammatory treatments in patients with TED. In comparison, research progress of PET is still restricted to disease diagnosis and monitoring, which might be mainly due to lack of sample size in PET examination (82, 83). Nevertheless, recent research has revealed the potential of Zirconium-89-rituximab PET/CT as a predictive marker for rituximab treatment response in TED (84, 85), which might provide insights for future research into further exploring the potency of PET in predicting anti-inflammatory treatment response.

### 4.3 DITI

DITI is a non-invasive technique that measures mid to long-wave infrared radiation emanating from human skin and converts it into temperature with a digitized image, forming a thermal map of the scene in false color (86). In the field of ophthalmology, previous studies have already demonstrated that DITI could indicate dry eye by reflecting temperature changes under closed chamber in closed and open position (87), laying foundation for further application of DITI in diagnosis and treatment of TED (88).

In the context of TED, several studies have demonstrated promising prospects of DITI imaging biomarker in assessing TED activity and predicting treatment response. By demonstrating significant difference in local temperature of medial conjunctiva, lateral conjunctiva, and lower eyelid, DITI has been proved with better performance than CAS in activity assessment (88, 89). In return, DITI complements limitations of CAS when clinical signs are subtle. Furthermore, in terms of predicting treatment response to IVGC, Shih et al. (90) developed a thermographic index-based prediction model by employing basal eye temperature measured by DITI as an objective indicator of TED inflammation. Compared to simple use of CAS, a combined model with higher predictive accuracy was developed based on mean temperature of specific points collected from various eye structures and gender (AUC=0.828 vs. 0.666), which was later verified by Dave et al. (91). However, the clinical application of DITI is currently limited, and its role in the prediction model of anti-inflammatory treatment response requires further validation.

In conclusion, although DITI is not as widely applied as other imaging techniques like MRI or nuclide imaging, imaging biomarkers derived from DITI has shown excellent performance in predicting treatment response to anti-inflammatory treatment in TED. The advantages lie in its ability to provide non-contact and ionizing radiation-free temperature measurements that enable convenient and safe identification of temperature changes at inflammatory sites. However, with limited attempts for exploration of DITI in TED in the past, it requires further research for validation of uniformity in infrared temperature acquisition and analysis in the future.

### 4.4 Other modes of imaging

CT and ultrasound examination are two other modes of imaging widely applied in TED. However, their application was restricted to TED diagnosis and monitoring, lacking in ability for predicting anti-inflammatory treatment response.

#### 4.4.1 CT

CT is a highly efficient, economical, and accurate detecting technique that provides precise reflection of the boundary between soft and hard tissues in the orbit. Common application of CT imaging biomarkers includes depiction of invasive growth of orbital tumors and extraocular muscle thickening (92, 93). However, in the field of TED, CT is less referable than MRI in anti-inflammatory treatment response prediction due to its shortage in reflecting soft tissue pathogenesis. Nocaudie et al. (87) has attempted to use CT indicators for predicting the treatment response to IVGC but failed. They concluded their failure to the superimposition of structures, which could lead to a reduction in contrast and fail to define a good quantitative index on planar images. On the other hand, in the surgical treatment for TED, CT imaging biomarkers turned out to be outstanding in predicting treatment efficacy. Based on CT quantitative indicators of orbital trigone width (P<0.001) and fat removal volume (P<0.001), Kitaguchi et al. (94) established a prediction model for treatment efficacy after orbital decompression surgery. Meanwhile, Sobti et al. (95) found that eye muscle thickness significantly correlated with the decrease of postoperative exophthalmos (P=0.043). Therefore, subsequent researchers may further explore the predictive ability of CT in more TED ophthalmic surgery scenarios. In the future, by incorporating radiomics, deep learning and other advanced techniques into CT image processing, CT could play a supplementary role than high cost, time-consuming MRI in treatment response prediction (92).

#### 4.4.2 Ultrasound examination

Due to its easy operation, low cost and non-radiological damage, ultrasound examination has been applied in TED diagnosis since the 1950s. Two types of ultrasound biomarkers commonly used include: A-type ultrasound and B-type ultrasound (96). A-type ultrasound features strong tissue specificity, which can accurately measure the thickness of extraocular muscles and reflect the inflammatory edema based on the echogenicity of extraocular muscles (97). B-type ultrasound is mainly used for observation of the morphology of orbital tissue, which assists in determining rectus thickness, detecting optic neuropathy and providing therapeutic observation (98). However, due to its intrinsic principles, the accuracy and repeatability of ultrasound examination is susceptible to multiple factors in TED (i.e., measurement angle, artifacts, tissue fibrosis or calcification, postoperative morphological changes, etc.) (99). Therefore, the application of ultrasound imaging biomarkers is still limited to TED examination, diagnosis and morphological monitoring, while prediction models for treatment response based on ultrasound technique are few (100).

## 5 Discussion

**Figure 1:**
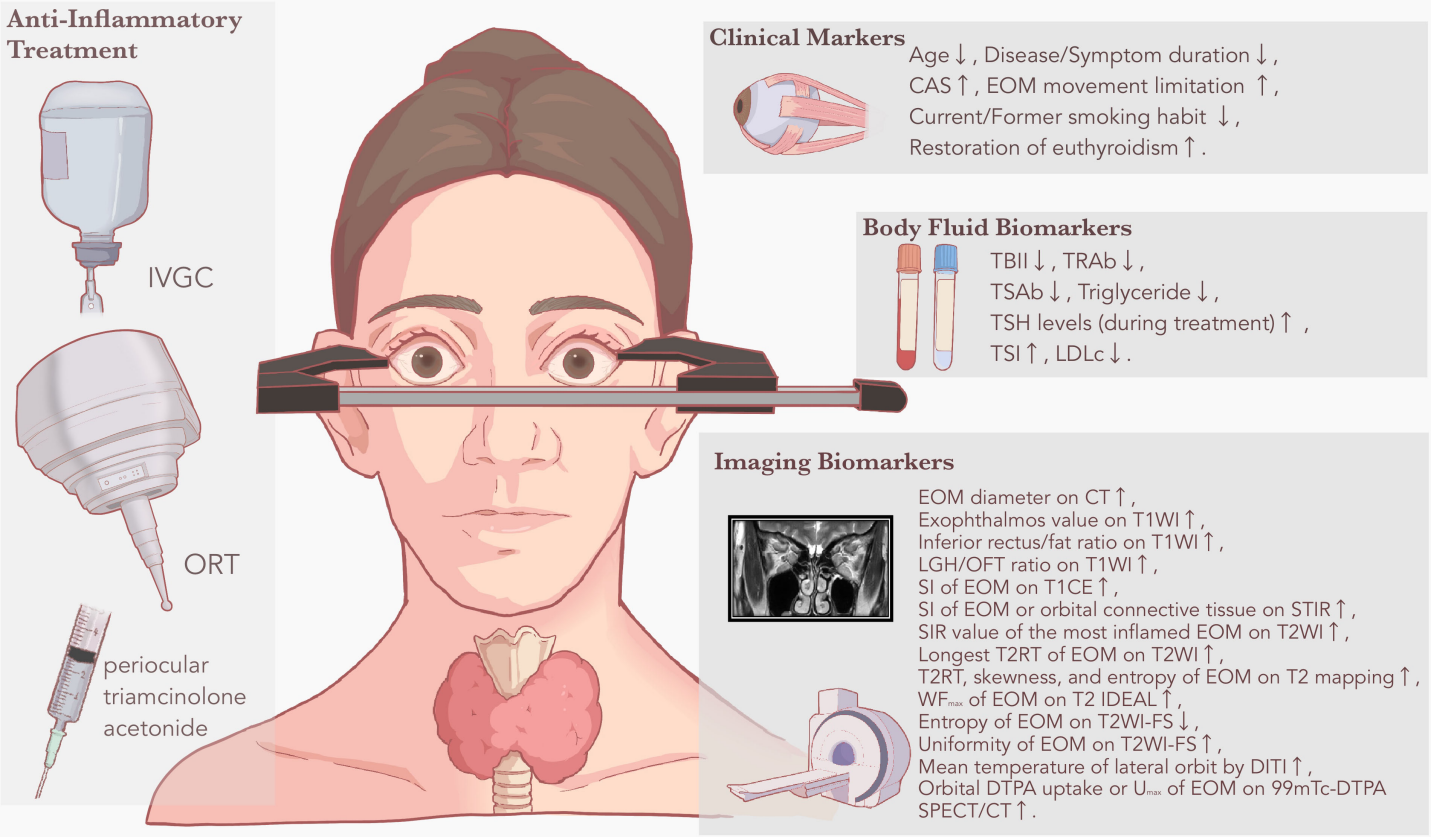
Clinical and biological markers for predicting anti-inflammatory treatment response in TED. Based on the literature review, common anti-inflammatory treatment methods identified with predictive markers or prediction models include IVGC, ORT, and periocular triamcinolone acetonide. Based on different derivations, predictive markers are displayed as clinical markers, body fluid biomarkers and imaging biomarkers, respectively. A rising arrow suggests that the predictive marker is positively related to treatment response to anti-inflammatory treatment, while a falling arrow suggests that the predictive marker is negatively related to treatment response to anti-inflammatory treatment. CAS, clinical activity score; CT, computed tomography; DITI, digital infrared thermal imaging; DTPA, diethylenetriaminepentaacetic acid; EOM, extraocular muscle; FS, fat-suppressed sequences; IVGC, intravenous glucocorticoid; LDLc, low-density lipoprotein cholesterol; LGH, lacrimal gland herniation; MRI, magnetic resonance imaging; OFT, orbital fat thickness; ORT, orbital radiation therapy; SI, signal intensity; SIR, signal intensity ratio; SPECT, single-photon emission computed tomography; STIR, short time of inversion recovery; T1WI, T1-weighted sequences; T2RT, T2 relaxation time; T2WI, T2-weighted sequences; TBII, thyrotropin-binding inhibitory immunoglobulin; TED, thyroid eye disease; TRAb, thyrotropin receptor antibody; TSAb, thyroid-stimulating antibody; TSH, thyroid-stimulating hormone; TSI, thyroid stimulating immunoglobulin; U_max_, maximum uptake ratio; WF_max_, maximum water fraction of extraocular muscle scintigraphy.

TED is a common autoimmune eye disease that has a well-established grading and staging system. However, due to the complexity of the disease itself and the lack of a unified rating method, the clinical application of TED prediction models for treatment response still faces challenges, including poor practical adaptation and unstable treatment effects. Therefore, there is a clinical need to search for a better association between pathogenesis and treatment methods. In this regard, biomarkers for depicting pathological progress can serve as the bridge. By summarizing predictive markers among current anti-inflammatory treatment research, this review classifies commonly used clinical and biological markers into three categories: clinical markers, body fluid biomarkers, and imaging biomarkers (**Figure 1**).

Each type of biomarker has its own merits and limitations in predicting the treatment response to anti-inflammatory treatment for TED. Clinical markers are easily accessible but may lack stability and accuracy. For better predictive accuracy, they should be combined with other type of predictive markers as a supplementary element. Body fluid biomarkers have relatively limited application due to their susceptibility to metabolic process, but tear biomarkers may overcome this shortcoming. As a product unique to the eye, tear biomarkers undergo less interference from body circulation, however, its lack of accessibility and high-quality evidence remain to be overcome in the future. Among imaging biomarkers, MRI performs best in diagnosing and treating TED by accurately capturing fibrous lesions and inflammatory status in the posterior part of the orbit. For enhancement of predictive accuracy, researchers have not only explored different MRI sequences, but also employed novel image processing technologies such as radiomics techniques. SPECT provides good predictive markers for anti-inflammatory treatment response, while PET currently performs unremarkably but holds potential for future advancements. DITI shows potential as an emerging approach for predicting treatment response in TED, mainly owing to its easy acquisition and unique clinical reflection. On the other hand, the application of other modes of imaging including CT and ultrasound is restricted to TED diagnosis and monitoring, with less satisfactory performance in prediction of anti-inflammatory treatment. Moreover, combination of different types of predictive markers has been discovered with better prediction ability. Some researchers have successfully combined imaging biomarkers with clinical markers (AUC=0.968) (64), others have attempted to combine body fluid biomarkers with clinical markers (AUC=0.915) (29), with both endeavors resulting in significantly improved predictive ability.

However, there still exist some obstacles in the research of treatment response to anti-inflammatory treatment. Common problems include different selection of evaluation criteria and evaluation timing, which is mainly due to lack of universality among research centers. For example, in the selection of evaluation timing, Ahn et al. (20) chose 3 months after treatment while Hu et al. (52) chose 6 months after treatment. When it comes to the selection of evaluation criteria, Zhou et al. (59) simply chose decrease in CAS by at least 2 points, while Wang et al. (65) chose a compound criteria with decrease in CAS by at least 2 points accompanied with at least one of the other improvements (i.e., decrease of proptosis ≥ 2 mm, decrease of lid width ≥2 mm, etc.). Such difference in evaluation would make it hard to compare the exact predictive value of different biomarkers among studies. Therefore, it is necessary to establish a unified evaluation standard for future treatment response prediction. Another communal problem lies in the lack of stratified analysis among different population groups. For instance, though age has already been demonstrated as a positive independent predictive marker for treatment response (20, 27), few studies have actually taken it as a stratification factor into their study design. Therefore, more specified classifying methods need to be employed, including more detailed classification according to different gender and age groups, different treatment stages, recovery status and etc.

In summary, recent years witnessed rapid development in the discovery of accurate predictive markers for treatment response to anti-inflammatory treatment in TED. Combination of predictive markers in different types, as well as research methods supported by innovative algorithm (101) or artificial intelligence (92), each new attempt has further enhanced the predictability of current models. However, we still need to clearly understand that current prediction models face common problems, including inconsistency of evaluation criteria and evaluation timing among centers, as well as heterogeneity among population groups. With continuous refinement in research methods, it is anticipated that predictive markers and prediction models for anti-inflammatory treatment response in TED will achieve wider clinical use for the benefits of more patients.

## Author contributions

HYZ: Conceptualization, Methodology, Writing – original draft. JF: Formal Analysis, Writing – original draft. JQ: Data curation, Visualization, Writing – review & editing. QH: Writing – review & editing. HFZ: Supervision, Validation, Writing – review & editing. XS: Supervision, Validation, Writing – review & editing.

## Glossary

**Table:**

ADC	apparent diffusion coefficient
BMI	body mass index
CAS	clinical activity score
CT	computed tomography
CXCL	CXC chemokine ligand
DITI	digital infrared thermal imaging
DTPA	diethylenetriaminepentaaceticacid
DWI	diffusion weighted imaging
EOM	extraocular muscle
EUGOGO	European group on Graves’ orbitopathy
FS	fat-suppressed sequences
fT4	free thyroxine
GO	Grave’s orbitopathy
IGF-1R	insulin-like growth factor 1 receptor
IDEAL	iterative secomposition of water and fat with echo asymmetry and least-squares estimation
IVGC	intravenous glucocorticoid
LDLc	low-density lipoprotein cholesterol
LGH	lacrimal gland herniation
MRI	magnetic resonance imaging
NPV	negative predictive value
OFT	orbital fat thickness
OR	odds ratio
ORT	orbital radiation therapy
PET	positron emission tomography
PPV	positive predictive value
QoL	quality of life
RANTES	regulated upon activation, normal T Cell expressed and presumably secreted
ROI	region of interest
SD	standard deviation
SI	signal intensity
SIR	signal intensity ratio
SPECT	single-photon emission computed tomography
T1WI	T1-weighted sequences
T2WI	T2-weighted sequences
TBII	thyrotropin-binding inhibitory immunoglobulin
TED	thyroid eye disease
TNF-α	tumor necrosis factor-α
TRAb	thyrotropin receptor antibody
TSH	thyroid-stimulating hormone
TSI	thyroid stimulating immunoglobulin
U_max_	maximum uptake ratio
WF_max_	maximum water fraction of extraocular muscle
XGBoost	extreme gradient boosting
99mTc	99m-Technetium
